# MicroRNA-219-5p Promotes Tumor Growth and Metastasis of Hepatocellular Carcinoma by Regulating Cadherin 1

**DOI:** 10.1155/2018/4793971

**Published:** 2018-05-15

**Authors:** Jing Yang, Yuan-Yuan Sheng, Jin-Wang Wei, Xiao-Mei Gao, Ying Zhu, Hu-Liang Jia, Qiong-Zhu Dong, Lun-Xiu Qin

**Affiliations:** ^1^Department of General Surgery, Huashan Hospital, Cancer Metastasis Institute, Fudan University, 12 Urumqi Road (M), Shanghai 200040, China; ^2^Institutes of Biomedical Sciences, Fudan University, 131 Dong An Road, Shanghai 200032, China

## Abstract

MicroRNAs play significant roles in the development of cancer and may serve as promising therapeutic targets. In our previous work, miR-219-5p was identified as one of the important metastasis-related microRNAs in HCC. Here we demonstrated that miR-219-5p expression was elevated in HCC tissues and was associated with vascular invasion and dismal prognosis. In multivariate analysis, miR-219-5p was identified as an independent prognostic indicator for HCC patients. Functional mechanism analyses showed that miR-219-5p promoted HCC cell proliferation and invasion in* in vitro*, as well as* in vivo*, tumor growth and metastasis in nude mice models bearing human HCC tumors. In addition, cadherin 1 (CDH1) was revealed to be a downstream target of miR-219-5p in HCC cells. In conclusion, miR-219-5p promotes tumor growth and metastasis of HCC by regulating CDH1 and can serve as a prognostic marker for HCC patients.

## 1. Introduction

Hepatocellular carcinoma (HCC) is one of the most common causes of cancer-related deaths worldwide, with high incidence of tumor recurrence and metastasis [1]. Identification of molecular markers plays a critical role in predicting the clinical outcome and promoting individual therapies for patients with HCC [2, 3].

MicroRNAs (miRNAs) have been implicated in regulation of pathogenesis of human tumors and could be potential biomarkers for diagnosis and prognosis [4, 5]. Recent studies have demonstrated that miRNAs participate in diverse human cancers processes including cell differentiation, proliferation, and apoptosis, as well as invasion and metastasis. For instance, miR-125 is a tumor suppressor that can decrease cell proliferation and metastasis through suppressing LIN28B expression in HCC [6], while miR-122a exerts tumor promoting effects on HCC by p53-dependent way [7]. Thus, cancer-specific miRNAs might be promising targets for cancer therapy [8].

Recently, miRNAs are demonstrated to function as critical regulators of cancer invasion and metastasis [9]. In our previous work, miR-219-5p is identified as one of the significant metastasis-related miRNAs in HCC [10]. However, little is known of the possible mechanism of miR-219-5p involved in HCC metastasis. In the present study, we found that miR-219-5p was upregulated in HCC tissues, was related to overall survival (OS) time of HCC patients, and promoted the proliferation and metastasis of HCC cells via downregulating CDH1. These results provide a clear understanding of the underlying mechanism by which miR-219-5p promotes HCC metastasis.

## 2. Materials and Methods

### 2.1. Clinical Tissue and Cell Culture

HCC tissues were obtained from patients who are treated with surgical resection in Huashan Hospital, Fudan University, and each patient had specific clinical-pathological information. Before surgical operations and collections of clinical tissues, all individuals wrote informed consent.

Human HCC cell lines Hep3B, Huh7, HepG2, MHCC-97H, and HCCLM3 were cultured in DMEM (Gibco) with 10% FBS. And they were propagated at 37°C in 5% CO_2_.

### 2.2. Cell Transfection

HepG2 and MHCC-97H cells were transfected with miR-219-5p mimic (50 nM) and miR-219-5p antagomir (400 pmol/ml) according to the manufacturer instructions. miR-219-5p mimic, antagomir, and their corresponding negative controls were purchased from Ribobio (Shanghai, China).

### 2.3. RNA Extraction and Quantitative Reverse Transcription-Polymerase Chain Reaction (qRT-PCR)

RNA of samples was obtained by TRIzol reagent (Invitrogen, USA). Then, we reversely transcripted RNA into cDNA according to the instruction of PrimeScript RT Master Mix and Mir-X miRNA First-Strand Synthesis Kit (TaKaRa, Shanghai, China). Next, cDNA was quantified by application of SYBR Premix Ex Taq II (TaKaRa) with gene-targeted or miR-specific primers. We applied the delta-delta Ct method to conduct quantification as well as calculation of the relative expression of each mRNA or miRNA. Primer sequences are listed in Table S1. Each sample was carried out three times.

### 2.4. Cell Proliferation Assay

The cell proliferation assay was conducted with Counting Kit-8 (CCK-8) (Tongren, Shanghai, China). 5000 cells (transfected with miR-219-5p mimic, miR-ctrl, miR-219-5p antagomir, antagomir NC) were planted in 96-well plates. Then 10% CCK-8 solution was added. The absorbance of each sample was assessed by a microplate reader set at 450 nM. Each sample was performed three times.

### 2.5. Cell Cycle Analysis and Apoptosis Assay

Cell cycle analysis was conducted with each sample fixed into 70% ethanol at 4°C. We added Propidium iodide (PI) and RNase to samples according to manufactures' instructions (Beyotime, Shanghai, China). After staining, cells were measured by flow cytometry (BD Bioscience, MA, USA). We analyzed results by Cell Quest software (BD Biosciences). Apoptosis assay was conducted by samples bound with Annexin V-FITC and 7-AAD according to the manufacturers' instructions (BD Bioscience). Then we analyzed samples by means of flow cytometry as described above. Each sample was replicated in triplicate.

### 2.6. Dual-Luciferase Reporter Assay

The wild-type sequence containing the predicted target sites of miR-219-5p in the 3′UTR of CDH1 mRNA was synthesized by JIELI corporation (Shanghai, China). We mutated the target sites from CUCCAC to GACCGA. After plasmid transfection, luciferase activities were assessed according to the manufacturer's instruction (Promega, USA). All samples were independently repeated three times.

### 2.7. Western Blotting

Samples were obtained with RIPA lysis buffer added with protease inhibitors. After quantification with bicinchoninic acid (BCA) assay (Weiao, Shanghai, China), we separated each protein through 10% SDS-PAGE and then moved them onto PVDF membranes (Millipore, USA). Then, samples were blocked with 5% nonfat milk. After incubation with primary antibodies against GAPDH and CDH1 (Cell Signaling Technology, Danvers, MA, USA) and secondary antibodies, protein levels were detected with ImageQuant™ LAS 4000 (GE Healthcare Life Sciences). Each sample was analyzed three times.

### 2.8. Cell Migration and Invasion Assay

The methods of cell migration and invasion assays were constructed as previously described [11].

### 2.9. Animal Model

Subcutaneous HCC model was established by injecting 5 × 10^6^ MHCC-97H cells (transfected with antagomir NC or antagomir miR-219-5p) into BALB/c nude mice (Shanghai SLAC Laboratory Animal Co.). And after 6 weeks, the tumor of each group was isolated. To establish in vivo tumor metastasis model, we transplanted tumors tissues (1~2 mm^3^) from the above subcutaneous HCC model to the livers of BALB/c mice. After 6 weeks, the tumors and lung tissues were obtained.

Tumor size was measured twice weekly with a calliper and the volume was calculated in mm^3^.

### 2.10. Statistical Analysis

Data was reported as mean ± SD. Data analysis was conducted by IBM SSPS Statistics Version 22. *X*^2^ and *t*-test were applied to measure differences between groups. Results were determined to be statistically significant when *P* < 0.05.

## 3. Results

### 3.1. miR-219-5p Upregulation Is Associated with Metastasis and Dismal Prognosis of HCC

**Figure 1:**
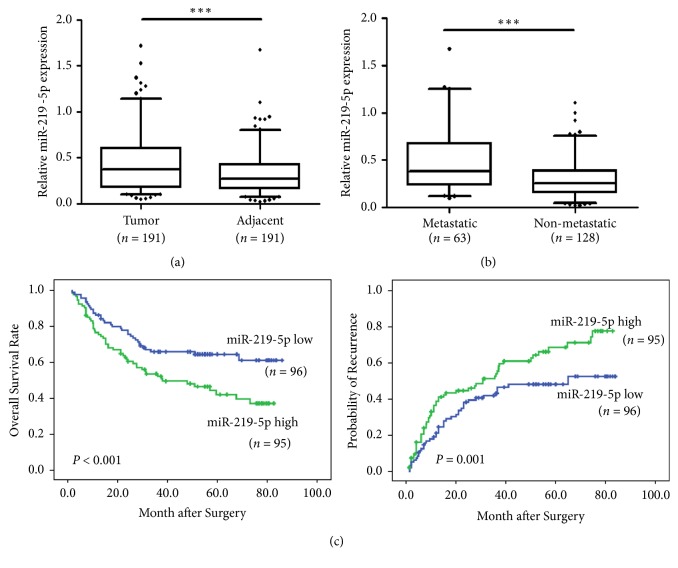
*The association of miR-219-5p upregulation with metastasis and prognosis of HCC.* (a) Relative expressions of miR-219-5p in 191 paired liver cancer tissues and paracancerous tissue samples. (b) The comparison of miR-219-5p levels between metastatic and nonmetastatic HCC tissues. Patients with high miR-219-5p level had a trend of worse overall survival (c) and significantly high recurrence rates compared with those with low miR-219-5p (d). Data are shown as mean ± SD. ^*∗∗∗*^*P* < 0.001 versus the control.

We analyzed the expression levels of miR-219-5p in 191 paired HCC tissues and corresponding noncancerous liver tissues by using qRT-PCR and found that miR-219-5p was significantly increased in HCC tissues compared with the nontumor liver tissues (*P* < 0.001) (Figure 1(a)). The expression levels of miR-219-5p were remarkably higher in HCC patients with metastasis in comparison to those without metastasis (*P* < 0.001) (Figure 1(b)). To further validate the role of miR-219-5p in HCC metastasis, we analyzed miR-219-5p in various HCC cell lines with different metastatic potentials and found that miR-219-5p levels in the HCC cells with high metastatic potentials were higher than those nonmetastatic cell lines (Supplementary Figure 1(A)). These results indicated that miR-219-5p upregulation is correlated with HCC metastasis.

Moreover, elevated miR-219-5p expression was found to be correlated with vascular invasion (*P* = 0.003) and worse differentiation (*P* = 0.011) of liver tumor, as well as severe liver cirrhosis (*P* < 0.001) (Table 1). Kaplan–Meier analysis showed that miR-219-5p overexpression was associated with poorer overall survival and higher recurrence rates of patients after curative HCC resection (Figure 1(c)). Univariate analysis showed that miR-219-5p, tumor size, tumor encapsulation, and vascular invasion were related to overall survival (OS) (Table 2); miR-219-5p, HBsAg, tumor size, vascular invasion, and tumor number were associated with HCC recurrence (Table 3). Multivariate analysis showed that miR-219-5p, vascular invasion, and tumor size were independent prognostic indicators for overall survival and tumor recurrence. Therefore, these results suggested that miR-219-5p upregulation can be a predictor of metastasis and dismal prognosis of HCC patients.

### 3.2. The Effects of miR-219-5p on In Vitro Proliferation and Invasion of HCC Cells

**Figure 2:**
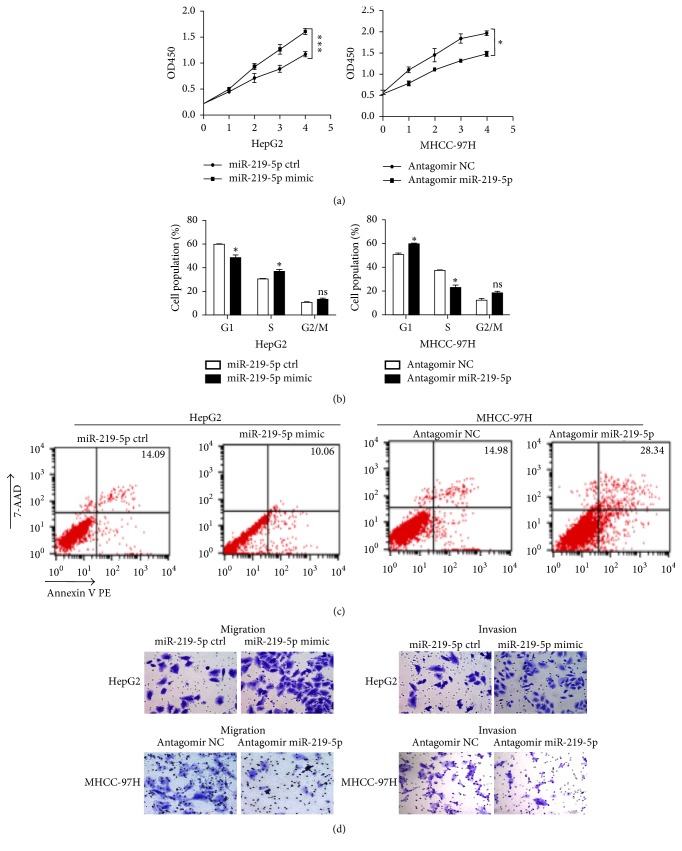
*The effects of miR-219-5p on in vitro proliferation and invasion of HCC cells.* (a) The alterations in cell proliferation of HepG2 cells after upregulation of miR-219-5p by mimic transfection (left) and MHCC-97H cells after knockdown of miR-219-5p by antagomir (right) were detected by CCK8 assay. (b) The cell cycle distribution of HepG2 and MHCC-97H cells after transfection with miR-219-5p mimic or antagomir. (c) Representative pictures of apoptosis of HepG2 and MHCC-97H cells after transfection with miR-219-5p mimic or antagomir detected by flow cytometry. (d) Migration and invasion of cells were determined by transwell assay in HepG2 and MHCC-97H cells treated with miR-219-5p mimic/antagomir and the corresponding negative control (magnification ×100). Data are shown as mean ± SD. ^*∗*^*P* < 0.05, ^*∗∗∗*^*P* < 0.001 versus the control.

To investigate the biological significance of miR-219-5p, we treated human HCC cell lines with miR-219-5p mimic or antagomir that would lead to different expression levels of miR-219-5p. Upregulation of miR-219-5p in HepG2, which had a low endogenous expression level, by miR-219-5p mimic induced significant increases in the abilities of proliferation (Figure 2(a); Supplementary Figure 2(A)). On the other hand, knockdown of miR-219-5p in MHCC-97H (with a high endogenous miR-219-5p level) by miR-219-5p antagomir (Supplementary Figure 2(A)) significantly inhibited the proliferation of cells (Figure 2(a)). What is more, the cell cycle distribution analysis showed that the cell number in G1 phase of HepG2 cells treated with miR-219-5p mimic was obviously decreased, and the cell number in S phase was increased compared with the ctrl. Cell cycle arrest at the G1 to S transition was found in MHCC-97H cells after treated with miR-219-5p antagomir (Figure 2(b); Supplementary Figure 2(B)). Furthermore, miR-219-5p mimic transfection significantly suppressed the apoptosis of HepG2 cells compared with ctrl, while miR-219-5p downregulation induced by miR-219-5p antagomir markedly promoted the apoptosis of MHCC-97H cells (Figure 2(c)). Next, we performed transwell assays to evaluate the invasion and migration abilities of HepG2 and MHCC-97H cells. Results showed that miR-219-5p upregulation significantly enhanced the migration and invasion abilities of HepG2, and miR-219-5p knockdown induced by miR-219-5p antagomir led to reduced number of migrated and invaded cells (Figure 2(d); Supplementary Figure 2(C)). Taken together, these data suggested that miR-219-5p can promote the proliferation, cell cycle transition of G1 into S phase, antiapoptotic potentials, and metastatic phenotype of HCC cells.

### 3.3. Effects of miR-219-5p on In Vivo Tumor Growth and Lung Metastasis of HCC Xenografts

**Figure 3:**
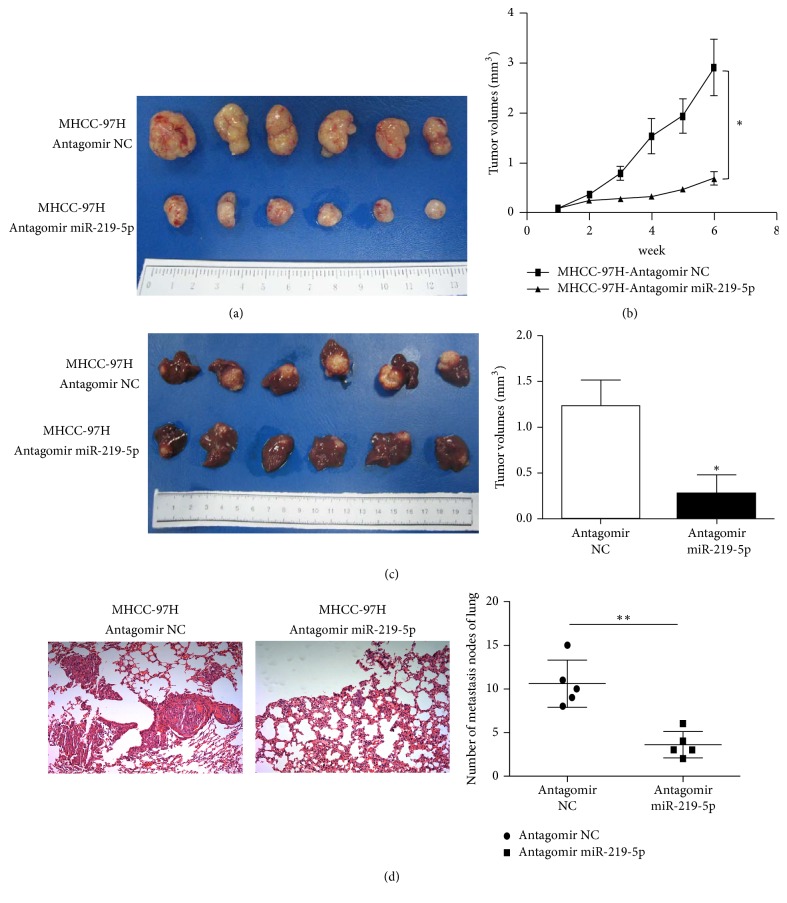
*Effects of miR-219-5p on in vivo tumor growth and lung metastasis of HCC.* (a, b) The differences in tumor size and volume of the subcutaneous implantation models of MHCC-97H cells after transfection with miR-219-5p antagomir or antagomir NC. (c) Comparison of the tumor volumes in the orthotopic implantation models of MHCC-97H cells after transfection with antagomir to knockdown miR-219-5p. (d) Representative images of lung metastasis (left) and comparison of the numbers of lung metastatic nodes in orthotopic implantation nude mice models of MHCC-97H cells after transfected with miR-219-5p antagomir or antagomir NC (magnification ×200). Data are shown as mean ± SD. ^*∗*^*P* < 0.05, ^*∗∗*^*P* < 0.01.

To further validate promoting roles of miR-219-5p in HCC progression, we established HCC xenografts models by subcutaneous implantation of MHCC-97H cells (transfected with miR-219-5p antagomir or antagomir NC). The average tumor volume of the miR-219-5p antagomir-treated group was obviously smaller than that of antagomir NC group (*P* < 0.05) (Figures 3(a) and 3(b)). To further validate its effect on the lung metastasis of HCC, the tumor tissues (1-2 mm^3^) were obtained from subcutaneous xenografts to establish orthotopic implantation models of nude mice. The average volume of orthotopic tumors in the miR-219-5p antagomir group was significantly smaller than that in the antagomir NC group (*P* < 0.05) (Figure 3(c)). Moreover, the total number of lung metastases in the miR-219-5p antagomir group was decreased compared with the antagomir NC (*P* < 0.01) (Figure 3(d)). These results suggested that miR-219-5p plays a crucial role in promoting* in vivo* tumor growth and lung metastasis of HCC.

### 3.4. CDH1 Is a Direct Target of microRNA-219-5p

**Figure 4:**
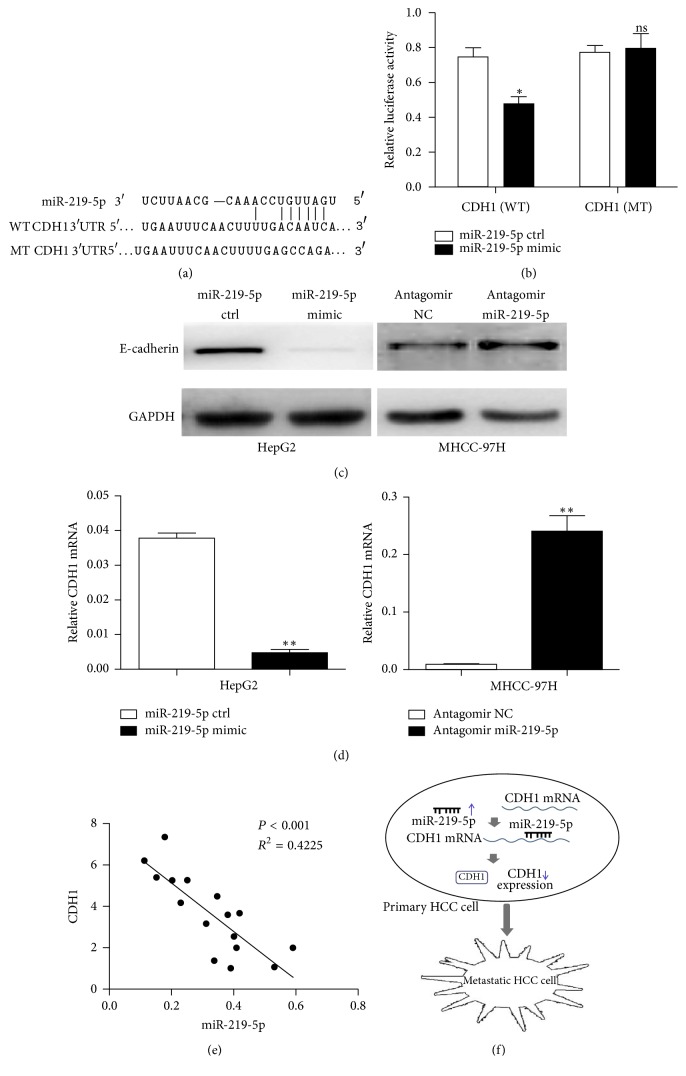
*CDH1 is identified as a downstream target of miR-219-5p.* (a) Sequences of CDH1 3′UTR and miR-219-5p according to the prediction of microRNA.org. Wild-type and mutated-type binding sequences of CDH1 3′UTR are shown. (b) Relative luciferase activity in HEK293T cells transfected with reporter vector containing wild-type or mutated-type binding sequence along with miR-219-5p mimic or negative control. CDH1 protein (c) and mRNA levels (d) in HCC cells treated with miR-219-5p mimic or antagomir. (e) Linear regression analysis between miR-219-5p and CDH1 in HCCs. (f) Working model for the role of miR-219-5p in regulation of CDH1 in HCC. Data are shown as mean ± SD. ^*∗*^*P* < 0.05, ^*∗∗*^*P* < 0.01.

Next, we searched for putative target genes of miR-219-5p in microRNA.org. We identified CDH1 as a direct target of miR-219-5p and the potential binding sequence in CDH1 3′UTR (Figure 4(a)). We carried out a dual-luciferase reporter assay to prove that CDH1 is a direct target of miR-219-5p. The reporter vector containing wild-type (CDH1-WT) or mutated-type binding sequence (CDH1-MT) was transfected into HEK293T cells along with miR-219-5p mimic or ctrl. Results showed that cotransfection of miR-219-5p with CDH1-WT, rather than with CDH1-MT, resulted in a significant decrease in luciferase activity compared with ctrl group (*P* < 0.05) (Figure 4(b)). To further validate the influence of miR-219-5p on CDH1, we overexpressed miR-219-5p in HepG2 cells and knocked down miR-219-5p in MHCC-97H cells, finding that miR-219-5p upregulation led to a significant decrease of CDH1 expression at both mRNA and protein levels (*P* < 0.01). And, miR-219-5p knockdown resulted in enhanced CDH1 expression (*P* < 0.01) (Figures 4(c) and 4(d)). Moreover, the linear regression analysis showed a negative relevance between miR-219-5p and CDH1 in HCC tissues (*R*^2^ = 0.4225; *P* < 0.001) (Figure 4(e)). These suggested that miR-219-5p is closely associated with negative regulation of CDH1 and CDH1 is a direct target of miR-219-5p.

## 4. Discussion

Metastatic relapse remains one of the major reasons for the dismal prognosis of HCC, which is a complicated process including cell adhesion, migration, and getting to target organs. Many molecules have been determined to be related to HCC metastasis [12]. However, the mechanism of HCC metastasis is not fully understood yet. Thus, characterizing the metastasis-related molecules and signaling pathways may provide more clues to the understanding of HCC metastasis. The clinical relevance and biological functions of miRNAs expression have been confirmed in various human solid tumors [13]. Thus, miRNAs were identified as superior molecular markers. Recently, an increasing number of studies have reported the indispensable roles of miRNAs in HCC [14–16]. In our previous study, miR-219-5p was found to be a promoter for HCC metastasis [10]. However, some studies demonstrated inconsistent results in other kinds of cancers. For example, miR-219-5p was reported to function as a tumor suppressor in colorectal and gastric cancers [17, 18]. The real reason is not clear. These results stimulate us to investigate the role of miR-219-5p in regulating aggressive phenotype of HCC cells.

In the present study, we found that miR-219-5p expression levels were remarkably upregulated in HCC tissues compared with the nontumor liver tissues, and high miR-219-5p levels were significantly associated with metastasis and dismal prognosis of HCC. Using gain- and loss-functional analyses, we found that miR-219-5p could promote in vitro proliferation, migration, and invasion of HCC cells. Furthermore, using loss-functional assays, we demonstrated that miR-219-5p promoted* in vivo* tumor growth and distal pulmonary metastasis of HCC. These provided more evidence to support that miR-219-5p is an important promoter for HCC growth and metastasis.

Another significant finding of the present study is that CDH1 is identified as a downstream target of miR-219-5p. CDH1, a suppressive oncogene, encodes the epithelial cell adhesion molecule, E-cadherin, which contributes to cell polarity and cell-cell adhesion [19–21]. Low expression levels of CDH1 were found to be correlated with aggressive clinicopathological factors and poor survival [22, 23]. Also, CDH1 inactivation resulted in the loss of cell-cell adhesion, which contributes to metastasis in a variety of tumors [24–26]. Furthermore, there is increasing evidence that multiple mechanisms are involved in the expression of CDH1, including epigenetic DNA methylation, somatic mutations, chromosomal deletions, and protein modification [11, 27, 28]. Recently, miRNAs have been determined to play gene-regulatory roles [28]. In this study, we found the following: (1) Bioinformatic analysis indicated that CDH1 can be a potential downstream target of miR-219-5p. (2) In a dual-luciferase reporter assay, cotransfection of miR-219-5p with CDH1 containing wild-type rather than mutated-type binding sequence resulted in a significant decrease in luciferase activity. (3) miR-219-5p upregulation led to a significant decrease of CDH1 expression; miR-219-5p knockdown resulted in enhanced CDH1 expression. (4) The linear regression analysis showed a negative relevance between miR-219-5p and CDH1 in HCCs. These indicated that miR-219-5p is closely associated with negative regulation of CDH1 and CDH1 is a direct target of miR-219-5p. Collectively, miR-219-5p promotes HCC growth and metastasis by downregulating CDH1 (Figure 4(f)).

In conclusion, these data suggest that miR-219-5p upregulation is an independent prognostic indicator for HCC patients. It plays an important role in promoting HCC growth and metastasis by downregulating CDH1. These provide more clues to develop novel strategies to combat HCC metastasis.

## Figures and Tables

**Table 1:** Relationship between miR-219-5p level and clinicopathologic features.

Variables	miR-219-5p expression		*P* value
	Low (*n* = 96)	High (*n* = 95)	
Sex			
Female	18	13	0.342
Male	78	82	
Age (years)			
≥50	32	30	0.796
<50	64	65	
HBV status			
Yes	94	43	0.599
No	9	15	
Cirrhosis			
Yes	16	55	**<0.001**
No	80	40	
AFP (ng/mL)			
>20	64	63	0.959
≤20	32	32	
Tumor size (cm)			
>5	40	43	0.616
≤5	56	52	
Tumor number			
Multiple	4	9	0.145
Single	92	86	
Tumor encapsulation			
Yes	52	45	0.347
No	44	50	
Vascular invasion			
Yes	31	51	**0.003**
No	65	44	
Tumor differentiation			
I~II	65	48	**0.011**
III~IV	31	47	

**Table 2:** Univariate and multivariate analyses of factors associated with overall survival (OS) inpatients with hepatocellular carcinoma (HCC).

Features	Overall survival			
	Univariate *P*	Multivariate		
		HR	95% CI	*P*
Sex				
Male versus female	0.537			NA
Age				
>50 versus ≤50	0.404			NA
HBsAg				
Positive versus negative	0.112			NA
AFP				
20 ng/ml versus ≤20 ng/ml	0.069			NA
Liver cirrhosis				
Yes versus no	0.307			NA
Tumor size				
>5 cm versus ≤5 cm	**<0.001**	2.620	1.675~4.099	**<0.001**
Tumor encapsulation				
Yes versus no	**0.017**	1.084	0.666~1.764	0.746
Tumor number				
Multiple versus single	0.065			
Vascular invasion				
Yes versus no	**0.001**	1.833	1.179~2.848	**0.007**
Tumor differentiation				
I~II versus III~IV	0.113			
miR-219-5p				
High versus low	**0.007**	**1.689**	**1.433~3.903**	**0.036**

**Table 3:** Univariate and multivariate analyses of factors associated with recurrence in patients with hepatocellular carcinoma (HCC).

Features	Recurrence			
	Univariate *P*	Multivariate		
		HR	95% CI	*P*
Sex				NA
Male versus female	0.331			NA
Age				NA
>50 versus ≤50	0.832			NA
HBsAg				NA
Positive versus negative	**0.043**	0.761	0.392~1.476	0.419
AFP				NA
20 ng/ml versus ≤20 ng/ml	0.615			NA
Liver cirrhosis				NA
Yes versus no	0.065			NA
Tumor size				NA
>5 cm versus ≤5 cm	**<0.001**	1.744	1.274~2.388	**<0.001**
Tumor encapsulation				NA
Yes versus no	0.671			NA
Tumor number				**NA**
Multiple versus single	**0.010**	1.579	0.751~3.318	0.228
Vascular invasion				NA
Yes versus no	**0.014**	1.870	1.205~2.902	**0.005**
Tumor differentiation				NA
I~II versus III~IV	0.539			NA
miR-219-5p				NA
High versus low	**0.014**	**1.663**	**1.072~2.577**	**0.023**

*Abbreviations*. HBsAg: hepatitis B surface antigen; HR: hazard ratio; CI: confidence interval.

## Supplementary Materials

Supplementary Materials Table S1: the primers listed were used for qPCR. Supplementary Figure 1: (A) miR-219-5p expressions in six liver cancer cell lines. GAPDH served as internal control. Data are shown as mean + SD. ^*∗*^*P* < 0.05, ^*∗∗*^*P* < 0.01. Supplementary Figure 2: the relative expression levels of miR-219-5p in HepG2 and MHCC-97H cells were examined after the cells were treated with miR-219-5p mimic, antagomir, or negative control (A) for 48 h using RT-qPCR. The analysis of distribution of cells with miR-219-5p mimic/antagomir was shown by histogram (B). The histogram of migration and invasion shows the mean ± SD of three independent experiments (C). Data are shown as mean + SD. ^*∗*^*P* < 0.05, ^*∗∗*^*P* < 0.01.
