# Supplementary material for: MicroRNA-219-5p Promotes Tumor Growth and Metastasis of Hepatocellular Carcinoma by Regulating Cadherin 1
Source: Biomed Res Int. 2018 May 15;2018:4793971. doi: 10.1155/2018/4793971 (PMC5976989; doi:10.1155/2018/4793971)

Supplementary Material

Table S1. The primers listed were used for qPCR.

Supplementary figure 1. (A) MiR-219-5p expressions in six liver cancer cell lines. GAPDH served as internal control. Data are shown as mean+SD. $*P < 0.05$, $**P < 0.01$.

Supplementary figure 2.

The relative expression levels of miR-219-5p in HepG2 and MHCC-97H cells were examined after the cells were treated with miR-219-5p mimic, antagomir or negative control (A) for 48 h using RT-qPCR. The analysis of distribution of cells with miR-219-5p mimic/antagomir was shown by histogram (B). The histogram of migration and invasion shows the mean ± SD of three independent experiments(C). Data are shown as mean+SD. $*P < 0.05$, $**P < 0.01$.

Table S1. The primer sequences used in qRT-PCR.

Gene	Sequence
GAPDH	5'-GCACCGTCAAGGCTGAGAAC-3'
GAPDH	5'-TGGTGAAGACGCCAGTGGA-3'
CDH1	5'-ATTCTGATTCTGCTGCTCTTG-3'
CDH1	5'-AGTCCTGGTCCTCTTCTCC-3'
miR-219-5p	5'-CGGTGATTGTCCAAACGCAATTC-3'

Supplementary 1

A

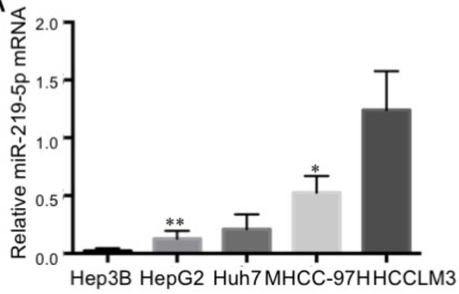

Supplementary 2

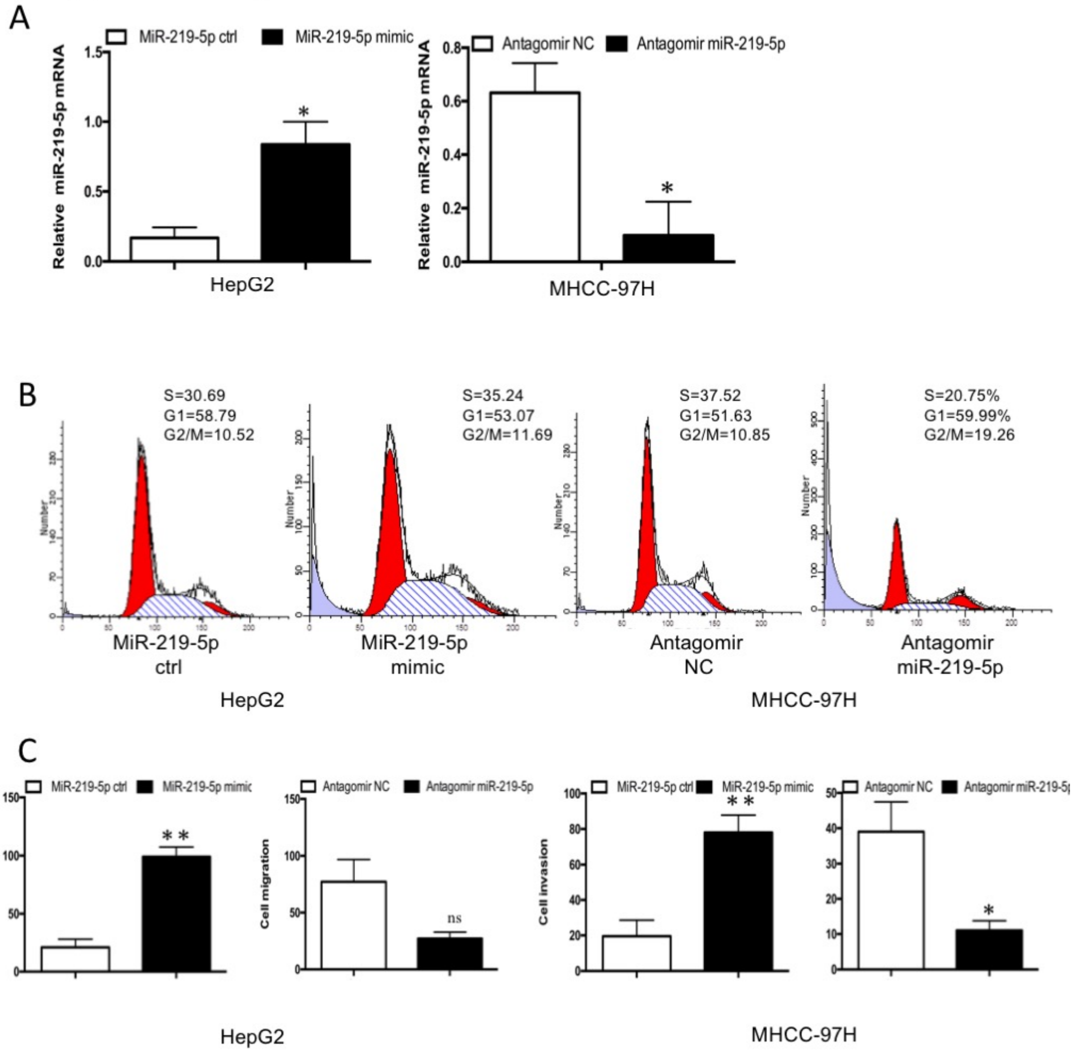

Supplement: Supplementary Materials — Table S1: the primers listed were used for qPCR. Supplementary Figure 1: (A) miR-219-5p expressions in six liver cancer cell lines. GAPDH served as internal control. Data are shown as mean + SD. ∗P < 0.05, ∗∗P < 0.01. Supplementary Figure 2: the relative expression levels of miR-219-5p in HepG2 and MHCC-97H cells were examined after the cells were treated with miR-219-5p mimic, antagomir, or negative control (A) for 48 h using RT-qPCR. The analysis of distribution of cells with miR-219-5p mimic/antagomir was shown by histogram (B). The histogram of migration and invasion shows the mean ± SD of three independent experiments (C). Data are shown as mean + SD. ∗P < 0.05, ∗∗P < 0.01. [file 4793971.f1.zip › supplementary material_BMRI_2233058.pdf]
