# Supplementary material for: MicroRNA-219-5p Promotes Tumor Growth and Metastasis of Hepatocellular Carcinoma by Regulating Cadherin 1
Source: Biomed Res Int. 2018 May 15;2018:4793971. doi: 10.1155/2018/4793971 (PMC5976989; doi:10.1155/2018/4793971)

**Supplementary Material**

**Table S1**. The primers listed were used for qPCR.

**Supplementary figure 1.** (A) MiR-219-5p expressions in six liver cancer cell lines. GAPDH served as internal control. Data are shown as mean+SD. **P* < 0.05, ***P* < 0.01.

**Supplementary figure 2.**

The relative expression levels of miR-219-5p in HepG2 and MHCC-97H cells were examined after the cells were treated with miR-219-5p mimic, antagomir or negative control (A) for 48 h using RT-qPCR. The analysis of distribution of cells with miR-219-5p mimic/antagomir was shown by histogram (B). The histogram of migration and invasion shows the mean ± SD of three independent experiments(C). Data are shown as mean+SD. **P* < 0.05, ***P* < 0.01.

**Table S1. The primer sequences used in** **qRT-PCR.**

| Gene | Sequence |
| --- | --- |
| *GAPDH* | 5'-GCACCGTCAAGGCTGAGAAC-3' |
| *GAPDH* | 5'-TGGTGAAGACGCCAGTGGA-3' |
| *CDH1* | 5'-ATTCTGATTCTGCTGCTCTTG-3' |
| *CDH1* | 5'-AGTCCTGGTCCTCTTCTCC-3' |
| *miR-219-5p* | 5'-CGGTGATTGTCCAAACGCAATTC-3' |


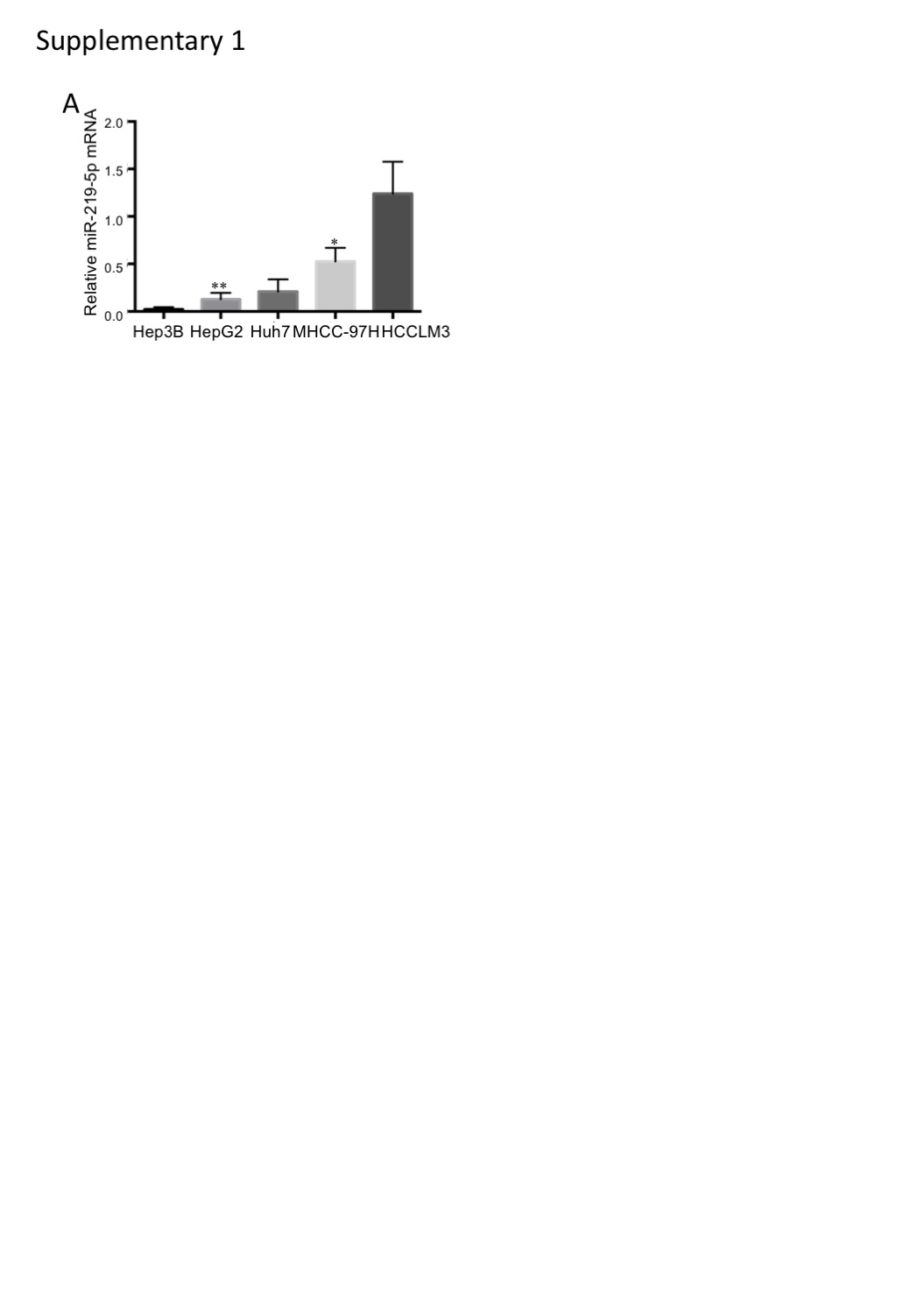


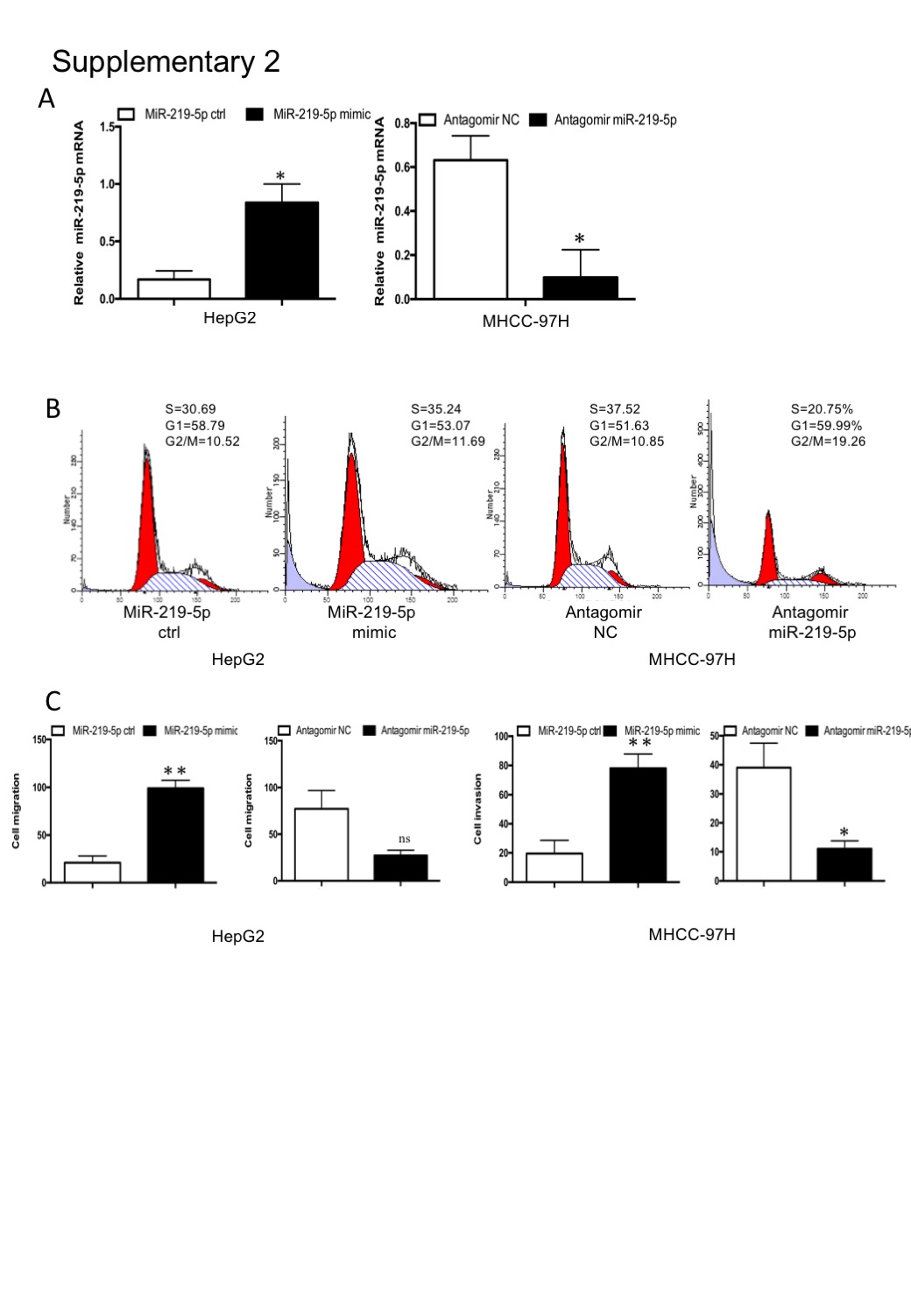

Supplement: Supplementary Materials — Table S1: the primers listed were used for qPCR. Supplementary Figure 1: (A) miR-219-5p expressions in six liver cancer cell lines. GAPDH served as internal control. Data are shown as mean + SD. ∗P < 0.05, ∗∗P < 0.01. Supplementary Figure 2: the relative expression levels of miR-219-5p in HepG2 and MHCC-97H cells were examined after the cells were treated with miR-219-5p mimic, antagomir, or negative control (A) for 48 h using RT-qPCR. The analysis of distribution of cells with miR-219-5p mimic/antagomir was shown by histogram (B). The histogram of migration and invasion shows the mean ± SD of three independent experiments (C). Data are shown as mean + SD. ∗P < 0.05, ∗∗P < 0.01. [file 4793971.f1.zip › supplementary material_BMRI_2233059.docx]
